# Compensation crisis related to the onsite adequacy evaluation during FNA procedures-Urgent proactive input from cytopathology community is critical to establish appropriate reimbursement for CPT code 88172 (or its new counterpart if introduced in the future)

**DOI:** 10.4103/1742-6413.71741

**Published:** 2010-10-18

**Authors:** Inderpreet Dhillon, Martha B. Pitman, Richard M. DeMay, Pamela Archuletta, Vinod B. Shidham

**Affiliations:** Department of Pathology, Wayne State University School of Medicine and Detroit Medical Center, Detroit, MI; 1 Massachusetts General Hospital, Harvard Medical School, Boston, MA; 2 University of Chicago, Chicago, IL

**Keywords:** CPT, FNA, Biospy, on site adequacy evaluation, 88172, Cytology, cytopathology

## Abstract

The confusion centered around appropriate use of the CPT billing code 88172 is addressed in the commentary from the Economic and Government Affairs Committee of the American Society of Cytopathology (ASC) who have written a timely commentary in this issue of Cytojournal, “Adequate Reimbursement is Crucial to Support Cost-Effective Rapid Onsite Cytopathology Evaluations”. Currently, lack of standardized use within and between pathology departments is stirring unhealthy practices of denying reimbursements for this critical and legitimate cytopathology service. This editorial discusses the important concerns raised in this commentary and recommends immediate corrective action. (See also Al-Abbadi MA, *et al*. Adequate reimbursement is crucial to support cost-effective rapid on-site cytopathology evaluations. CytoJournal 2010;7:22)

In response to confusion centered around appropriate use of the CPT billing code 88172 pertaining to immediate cytological evaluation, Al-Abaddi, *et al*. of the Economic and Government Affairs Committee of the ASC have written a timely commentary in this issue of Cytojournal, “Adequate Reimbursement is Crucial to Support Cost-Effective Rapid Onsite Cytopathology Evaluations”.[1] The October 2009 publication of the National Coding Corrective action policy manual,[2] attempting to clarify the parameters surrounding the appropriate use of the 88172 fee code, has been met with a lack of standardized use within and between pathology departments, and reimbursements for legitimate pathology services have reportedly been denied. Multiple important points are raised in this commentary.[1]

Application of CPT code 88172 was reported in the September, 2006 issue of *CAP Today*, where specifics were addressed on when and how to use the code. It was stated that code 88172 may be used as many times as a pathologist is asked to assess adequacy, but each application of the fee code requires proper documentation of each interpretation in the report.[3] However, lack of well-defined guidelines has allowed some carriers to deny appropriate reimbursement for these codes and the time-consuming service provided. Adequacy evaluation of each pass of any FNA is analogous to the frozen sectioning scenario with deeper sections of the same frozen block as well as additional tissue from the same specimen submitted for frozen section. Professional time and skilled interpretations of complex pathological interpretations, whether on multiple frozen sections or multiple immediate interpretations of an FNA, should be appropriately compensated [Table 1].

**Table 1:** Comparative reimbursement RVUs for onsite FNA adequacies, frozen section and touch prep.

*CPT Code*	*SERVICE*	*Time*	*RVU*	
88172	On site adequacy evaluation of FNA	35-56 min	Carrier A	Carrier B
			0.83	1.1
88331	First tissue block with frozen section(s) single specimen	10-20 min	1.7	2.2
88332	Each additional tissue block with frozen section(s)	10-20 min	0.82	1.07
88333	Cytological examination (i.e. touch prep) First area	10-15 min	1.7	2.2
88334	Cytological examination (i.e. touch prep) Each additional area	10-15 min	1.02	1.4

RVU, Relative value unit; FNA, fine needle aspirate[4]

It is widely accepted that immediate adequacy evaluation greatly reduces the cost of patient care.[5] Onsite adequacy evaluation also provides interactive real-time communication of information including appropriate tissue triage recommendations for ancillary tests such as flow cytometry, EM, cytogenetics, etc. This directly impacts clinical management during the critical diagnostic phase while the lesion can still be sampled readily.[6] Any compromise of this step will adversely affect the ultimate cost and quality of patient care [Figure 1]. Studies have reported the increase in diagnostic yield due to onsite adequacy evaluation with an obvious benefit to patient care [Figure 1].[7–10] Inability to provide onsite adequacy services would lead to increased cost due to an increased number of repeat procedures with resultant increase in patient morbidity and suboptimal care. Improper compensation practices disproportionate to the time and resource investment have already been pushing this service into disfavor by many pathology departments due to cost of providing this support.

**Figure 1:**
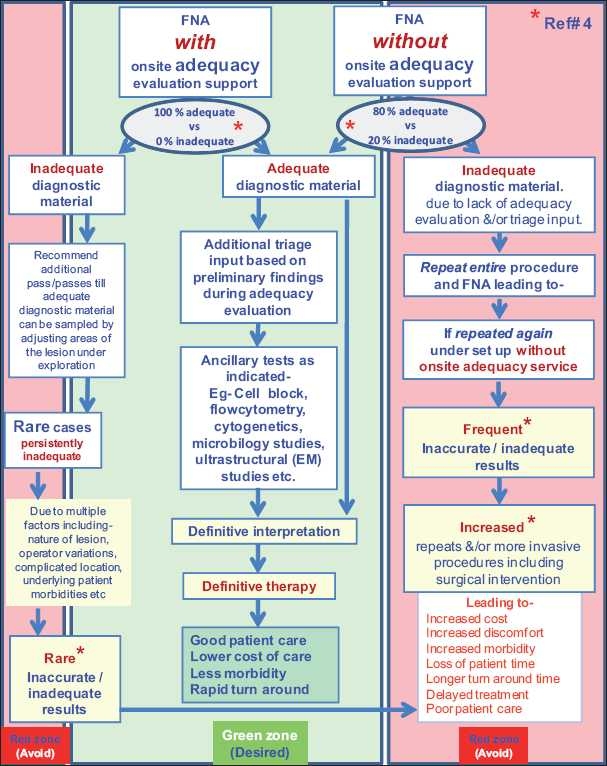
Comparative scenario in the evolution of patient care involving FNA with onsite adequacy versus without onsite adequacy.

Published literature criticizing the recent trends in compensation practices for the cytopathology services in this endeavor are relatively limited. In the study by Layfield *et al*, the time spent on various FNA adequacy evaluations was similar to that at Wayne State University Hospitals and ranged from 35 to 56 minutes with time cost exceeding compensation by $40-50 per procedure.[7] They concluded that intraprocedural consultations by the cytopathologist for onsite adequacy evaluation of FNA procedures are compensated insufficiently by the CPT code 88172 even with the use of a separate 88172 CPT code for each FNA pass and independent immediate interpretation. The payment may be adequate if the cytopathologist personally performs the aspirate with immediate onsite evaluation.[7] Wang *et al*., addressing the cost effectiveness of adequacy assessment with respect to thyroid FNAs,[9] reported that assessment increases the diagnostic yield of thyroid FNAs but at a tremendous expense to cytology service in time.[9]

At Wayne State University Hospitals, there is a fee code for each pass which is billed on separate lines using the modifier 26/76. However, reimbursement using modifier 26/76 in the current environment is unpredictable, as experienced by a few institutions with some insurance carriers, and periodically shared at discussions in the ASC listserv. Interestingly, some payers state that only one 88172 fee code can be billed per day. Lack of clear consensus or guidelines has led to a deteriorating trend in this component of patient care over time. In general, the insurance carriers lack insight into the technical aspects of the FNA adequacy process to correct this detrimental trend. Publications such as the current commentary from the Economic and Government Affairs Committee of the ASC, published here in *“open access”* for easy access to the public including policy makers, are critical and healthy trends to address this issue before it gets worse. We applaud the Economic and Government Affairs Committee of ASC for their initiative in this matter by publishing their commentary in the public domain.

It is important to note that the evaluation of different types of specimens by a variety of approaches also impacts the cost analysis. For example, performing pancreatic FNAs are more time consuming than performing FNAs of more easily accessible areas such as the thyroid, which requires a much longer time for onsite adequacy assessment with multiple passes.[11] It would be prudent that compensation for the onsite adequacy evaluation be adjusted with appropriate modifiers for the procedures that routinely require a longer time due to complexity such as with cytotechnologist (CT)-guided, ultrasound-guided, bronchoscopic or endoscopic FNAs and other factors [Table 2]. Proper compensation would encourage pathologists to devote the time for this critical function and may spur innovation such as the application of telecytopathology for remote immediate adequacy assessment.[12,13]

**Table 2:** Hypothetical reimbursement for onsite FNA adequacies after considering complexities and time factor.

*Onsite adequacy evaluation*	*Nature of pass(es)*	Suggested level of relative compensation code related to original CPT code of 88172 *	
		*X*	*Y*
		*For adequacy by Cytopathologist/cytopathology-trained pathologists*	*For adequacy by Cytotechnologists with cytopathologist/cytopathology-trained pathologists available for consultation*
FNA of superficial *lesions* with average complexity and time requirement	First pass	A. 88172- modifier 26 (Global)	M. 88172 TC-ADQ
	Additional pass(es)	B. 88172- with modifier 26/76 (Global)	N. 88172 TC-ADQ with modifier ‘a’
FNA of deeper *lesions* under image guidance such as US guided thyroid FNA	First pass	C. 88172- modifier 26+ (Global)	O. 88172 TC-ADQ+
	Additional pass(es)	D. 88172 with modifier 26/76+ (Global)	P. 88172 TC-ADQ + with modifier ‘a’
FNA of deeper lesions with complex procedures needing longer time, such as EUS-FNA, transbronchial FNA, intraoperative FNA	First pass	E. 88172-modifier 26++ (Global)	Q. 88172 TC-ADQ++
	Additional pass(es)	F. 88172 with modifier 26/76++ (Global)	R. 88172 TC-ADQ++ with modifier ‘a’

		***Illustration using numbers as percent: If basic compensation for CPT 88172 is 100%.***	
		‘A’ is 100%, ‘B’ may be 80% × n	‘M’ may be 80%, ‘N’ may be 60% × n
		‘C’ should be 120%, ‘D’ may be 100% × n	‘O’ should be 90%, ‘P’ may be 80% × n
		‘E’ should be 140%, ‘F’ may be 100% × n	‘Q’ should be 100%, ‘R’ may be 90% × n

Global is PC and TC combined together; PC, professional component; TC, technical component.,

+ indicates higher compensation and

++ indicates incrementally higher compensation more than just^+^, a, additional pass;

* To avoid complexity and simplify the coding, new CPT codes may be introduced with above principle.,

n = number of additional passes

Another issue of significance to be highlighted here is the role of the CT in onsite adequacy evaluation (not interpretation) under the supervision of the pathologist (who is available for consultation as needed). The CT has a definite role to play in the settings when pathologists are not available for onsite FNA services but can provide supervision with availability for direct input as indicated. In the current situation, 88172TC is not considered a stand-alone fee code without an associated 88172PC.[3] If a CT performs onsite adequacy under such conditions, it should be compensated by modified CPT 88172 (higher compensation than 88172TC included in global component). The modified 88172 for example may be 88172TC-ADQ [Table 2]. However, compensation for adequacy evaluations performed by CTs have different problems with additional ambiguity. Alsohaibani *et al*. showed that onsite FNA adequacy evaluation by CTs had an increased diagnostic yield compared with blind FNAs (77% versus 53%, respectively).[10] This approach would be a definite help in many institutions with limited availability of pathologists for onsite adequacy. The worst case scenario would be limitation or cessation of such services in the long run. Even in the academic settings with relatively less emphasis on the cost component due to the teaching value of the FNA adequacy exercise, currently there is an increasing reluctance to provide this support. A standard of practice across the board allowing separate billing for onsite adequacy of each pass of FNA is pivotal to prevent the potential debacle of this important service in patient care. This component is crucial for continued savings in overall patient care cost with better care and less morbidity. Given this fact, our clinical colleagues dependent on this support would agree that it is imperative that we proactively advocate the right approach. We look forward to the upcoming guidelines by the Center for Medical Services and strongly recommend that the cytopathology community let their voice be heard in the open public forum regarding this issue.
